# Association between islet β-cell function and time in range in patients with type 2 diabetes mellitus: a retrospective cross-sectional study

**DOI:** 10.3389/fendo.2026.1901834

**Published:** 2026-08-05

**Authors:** Ningning Li, Ling Lv, Zhaoyang Zhang, Yanjun Wang

**Affiliations:** Department of Endocrinology, The Second Hospital of Jilin University, Changchun, China

**Keywords:** beta-cell function, continuous glucose monitoring (CGM), HOMA2-β, time in range (TIR), type 2 diabetes

## Abstract

**Objective:**

Impaired islet *β*-cell function is the core mechanism underlying type 2 diabetes mellitus (T2DM). This study investigated the association between islet *β*-cell function and time in range (TIR) in patients with T2DM, providing a reference for individualized clinical treatment.

**Methods:**

This retrospective cross-sectional observational l study included 1,160 patients with confirmed T2DM. Participants underwent continuous glucose monitoring (CGM). TIR >70% was defined as achieving the glycemic target. Subjects were divided into three groups based on TIR levels (<70%, 70%–84%, and ≥85%). The *β*-cell function index *HOMA*2–*β* was calculated using the Homeostasis Model Assessment 2 as the core research indicator.

**Results:**

The high TIR group had significantly higher HOMA2-*β* (P < 0.001). Multiple linear regression analysis showed a robust positive correlation between HOMA2-*β* and TIR after adjusting for covariates (*β* = 0.402, 95% CI: 0.358–0.447, P < 0.001). Restricted cubic spline (RCS) analysis revealed a significant nonlinear relationship between HOMA2-*β* and TIR (P for nonlinearity < 0.001). TIR increased with rising HOMA2-*β*, but the rate of increase gradually slowed. Specifically, when HOMA2-*β* increased from approximately 10% to 100%, the predicted TIR rose rapidly from about 38% to approximately 96%, representing an increase of 58%. Sensitivity analysis using HOMA2-*β* calculated from fasting C-peptide instead of fasting insulin yielded consistent conclusions.

**Conclusion:**

A significant, nonlinear association exists between islet *β*-cell function *HOMA*2–*β* and TIR. TIR levels increase progressively with higher HOMA2-*β*. Although the cross-sectional design precludes causal inference, these findings provide important reference values for clinical decision-making.

## 1 Introduction

Type 2 diabetes mellitus (T2DM), as one of the chronic diseases with the highest global incidence, has become a major public health challenge. According to the International Diabetes Federation (IDF), the estimated global prevalence of diabetes mellitus (DM) among adults aged 20–79 years was 10.5% in 2021, and it is projected to rise to 12.2% by 2045 (1). China currently has the largest number of people with diabetes in the world, accounting for more than one-quarter of the global diabetic population (2). It is estimated that by 2050, the prevalence of diabetes in China will reach as high as 29.1% (3), posing a major challenge to the development of a “Healthy China” initiative in the future.

Pancreatic *β*-cells are terminally differentiated cells, and their progressive dysfunction is a core mechanism underlying the progression of type 2 diabetes mellitus (T2DM). Therefore, early identification of abnormal pancreatic *β*-cell function and its declining trajectory is of great significance for the precise prevention and treatment of T2DM.

Blood glucose monitoring is the cornerstone of T2DM management. Traditional monitoring methods primarily rely on glycated hemoglobin (HbA1c) measurement and capillary finger-prick blood glucose testing. HbA1c reflects the average blood glucose level over the previous 2–3 months, with a recommended control target of <7% (53 mmol/mol) (3–5), and is a classic indicator for assessing long-term glycemic control. However, HbA1c cannot effectively capture short-term glycemic variability. Although fingerstick blood glucose testing can provide real-time single-point glucose values, it requires frequent invasive procedures. Moreover, neither method can fully characterize the patterns of daily glycemic fluctuations. Related studies have shown that even patients with well-controlled HbA1c may still have inadequate day-to-day glycemic control (6).

The continuous glucose monitoring system (CGMS) addresses the limitations of traditional monitoring methods (7). Continuous glucose monitoring (CGM) using a subcutaneous sensor continuously measures interstitial fluid glucose concentrations, indirectly reflecting dynamic changes in blood glucose. It is now widely used in the glycemic management of patients with diabetes. CGM records glucose data every 1–5 minutes, generating a continuous and comprehensive daily glucose profile along with retrospective analysis reports, thereby providing complete information on glucose fluctuations. The various monitoring metrics derived from CGM—such as time in range (TIR, 3.9–10.0 mmol/L), time above range (TAR), time below range (TBR), glycemic coefficient of variation (CV), and mean blood glucose (MBG)—offer richer dimensions for assessing glycemic control. Among these, TIR is a key indicator, with a recommended control target of TIR > 70% (8). Furthermore, studies have shown that TIR is closely correlated with HbA1c (9, 10), and TIR has become an important target for evaluating the effectiveness of glycemic control (8).

In recent years, a large number of clinical studies have confirmed that TIR is closely associated with various chronic complications of T2DM (11–15). Studies have shown that TIR is negatively correlated with cognitive dysfunction (16, 17), cardiovascular injury (18), osteoporosis (19), metabolic dysfunction-associated fatty liver disease (20), and sarcopenia (21) in elderly patients with T2DM. Furthermore, TIR alone can serve as an effective predictor of carotid artery plaque (22–24), diabetic kidney disease (25–32), and diabetic peripheral neuropathy (33–35), and is also closely related to the severity of retinopathy (36–38) and cardiac autonomic neuropathy (39) in T2DM patients. Based on current evidence, higher TIR is associated with a lower risk of complications.

Current research on TIR has not only focused on its association with various complications (11, 40), but recent evidence has also begun to elucidate the direct relationship between *β*-cell function and TIR in patients with T2DM. Ye et al., in a study of 455 Chinese patients with type 2 diabetes, confirmed a positive correlation between TIR and HOMA-*β*; specifically, each 10-unit increase in HOMA-*β* was associated with a 10% increase in the probability of achieving TIR > 70% (41). Zhang et al. found that *β*-cell dysfunction, assessed by the insulin secretion-sensitivity index-2 (ISSI2), was significantly negatively correlated with TIR (42). Yang et al. reported that TIR is an independent protective factor for *β*-cell function, suggesting a possible bidirectional relationship between the two (32). However, these studies have mainly focused on small-scale linear associations, and no study has systematically explored potential nonlinear relationships in a large-scale cohort. Therefore, the present study aims to investigate the association between pancreatic *β*-cell function and TIR, and to explore whether a nonlinear relationship exists, thereby providing a more nuanced understanding for individualized glycemic management.

## 2 Methods

This retrospective cross-sectional observational study was reported in accordance with the Strengthening the Reporting of Observational Studies in Epidemiology (STROBE) guidelines. As a retrospective observational study based on inpatient electronic medical records, no prospective patient recruitment was performed.

### 2.1 Study population and design

This retrospective cross-sectional study initially included 1,204 patients diagnosed with T2DM (Diagnosis of T2DM was based on the 2023 WHO diagnostic criteria (fasting plasma glucose ≥7.0 mmol/L, or 2-hour postprandial glucose≥11.1mmol/L, or HbA1c≥6.5%)) (43) who were hospitalized and treated at the Department of Endocrinology, Yatai Campus of the Second Hospital of Jilin University, between Eligible cases were consecutively enrolled from July 1, 2024 to November 30, 2025. All patients underwent continuous glucose monitoring. Based on the study objectives, the exclusion criteria were as follows: 1) age <18 years; 2) Use of insulin or sulfonylureas within the previous six months, or thiazolidinediones (TZDs) (to avoid potential interference with the assessment of *β*-cell function); 3) other types of diabetes mellitus; 4) severe hepatic or renal impairment; 5) severe autoimmune diseases; 6) use of glucocorticoid therapy; 7) severe systemic diseases; and 8) recent major surgery. Patients treated with glucagon-like peptide-1 receptor agonists (GLP-1 RAs), dipeptidyl peptidase-4 inhibitors (DPP-4i), or sodium-glucose cotransporter 2 inhibitors (SGLT2i) were retained in the full cohort. This inclusion strategy was determined based on pharmacological mechanisms: GLP-1 RAs and DPP-4i exert glucose-dependent insulinotropic effects, with minimal impact on fasting insulin levels under euglycemic conditions, and thus do not cause persistent systematic bias in HOMA2-*β* calculation. SGLT2i lower blood glucose via urinary glucose excretion without direct effects on pancreatic *β*-cell function or fasting insulin levels, and therefore do not interfere with basal *β*-cell function assessment. GLP-1 RA users accounted for 9.6% (111 patients) of the total study population. Considering that treatments and adjustments to hypoglycemic regimens during hospitalization could induce glucose fluctuations, only the average CGM data from the three days prior to admission were selected to assess baseline glycemic control. After excluding 44 patients with incomplete data, 1,160 patients were included in the final analysis. Demographic and clinical data were collected via the inpatient electronic medical record system, including age, sex, diabetes duration, body mass index (BMI), history of hypertension, family history, smoking and drinking history, history of coronary heart disease, treatment history, and laboratory indicators at admission. BMI was calculated as weight in kilograms divided by the square of height in meters *kg*/*m*^2^. Hypertension history and smoking history were treated as ordinal variables. Treatment history was categorized into untreated, metformin monotherapy, metformin combined with other oral agents and other treatments (glucagon-like peptide-1 receptor agonists (GLP-1 RAs), dipeptidyl peptidase-4 inhibitors (DPP-4i), or sodium-glucose cotransporter 2 inhibitors (SGLT2i); none of these patients used insulin, sulfonylureas, or thiazolidinediones.). Data extraction, quality control and statistical analysis were completed between January and March 2026.The baseline characteristics of the participants are detailed in **Table 1**.

**Table 1:** Participants characteristics.

Characteristic	Value
N	1160
Male sex, n (%)	695 (59.90)
Age (year)	52.3 ± 12.76
BMI(kg/m2)	26.25 ± 3.96
Duration of diabetes (year)	3 (0.5-7.0)
History of smoke, n (%)	
NO	862 (74.30)
<10years	97 (8.40)
≥10years	201 (17.30)
History of drink, n (%)	282 (24.30)
History of coronary heart disease, n (%)	183 (15.80)
Family history of diabetes, n (%)	505 (43.50)
History of hypertension, n(%)	
non-hypertensive subjects	662 (57.10)
Grade 1 hypertension subjects	95 (8.20)
Grade 2 hypertension subjects	164 (14.10)
Grade 3 hypertension subjects	239 (20.50)
Treatment history,n (%)	
Untreated	363 (31.30)
Metformin monotherapy	345 (29.70)
Metformin and other oral hypoglycemic agents	172 (14.80)
Other treatment regimens	280 (24.14)
HbA1c at admission (%)	8.00 (6.90-9.60)
FPG at admission (mmol/L)	8.56 (7.10-11.10)
Fasting C-peptide(ng/mL)	1.87 (1.36-2.53)
Fasting insulin(μU/mL)	9.82 (6.47-14.43)
1-hour postprandial C-peptide(ng/mL)	3.24 (2.24-4.57)
1-hour postprandial insulin(μU/mL)	30.99 (19.96-47.48)
2-hour postprandial C-peptide(ng/mL)	4.29 (2.94-6.18)
2-hour postprandial insulin(μU/mL)	30.10 (23.25-53.63)
Triglyceride(mmol/L)	2.13 (1.49-3.42)
Cholesterol(mmol/L)	5.02 (4.26-5.83)
High-density lipoprotein (mmol/L)	1.18 ± 0.35
Low-density lipoprotein(mmol/L)	2.94 ± 0.94
Serum creatinine(μmol/L)	65.49 ± 20.85
Serum uric acid(μmol/L)	335.29 ± 93.32
Urea(mmol/L)	6.05 ± 1.73
eGFR (ml/min/1.73m2)	102.50 ± 17.19
ALT (U/L)	25 (18-40)
AST (U/L)	21 (17-29)
CV	22.81 ± 5.61
LAGE	11.06 ± 3.15
GMI	6.9 (6.5-7.5)
MAGE	4.9 (3.8-6.2)
SDBG	2.1 (1.7-2.6)
MBG	8.73 ± 1.87
Non-alcoholic fatty liver,n (%)	995 (85.80)
Carotid artery plaque,n (%)	905 (78.0)
Lower extremity arterial plaque,n (%)	621 (53.50)
HOMA2-β(FINS)	41% (24.2%-63.3%)
HOMA2-IR(FINS)	1.49 (1.02-2.165)
HOMA2-β(FCP)	43.4% (28%-63.4%)
HOMA2-IR(FCP)	1.68 (1.20-2.40)

Baseline characteristics of the study population. Data are presented as mean ± SD for normally distributed continuous variables, median (IQR) for non-normally distributed continuous variables, and n (%) for categorical variables.

### 2.2 Clinical and laboratory measurements

Laboratory data included fasting plasma glucose (FPG), HbA1c, fasting C-peptide (FCP), fasting insulin (FINS), 1-hour postprandial C-peptide and insulin, 2-hour postprandial C-peptide and insulin, triglycerides (TG), total cholesterol (TC), high-density lipoprotein cholesterol (HDL-C), low-density lipoprotein cholesterol (LDL-C), alanine aminotransferase (ALT), aspartate aminotransferase (AST), serum uric acid (SUA), serum creatinine (SCr), blood urea nitrogen (BUN), estimated glomerular filtration rate (eGFR), lower extremity arterial ultrasound, carotid arterial ultrasound, and the presence of fatty liver. Fasting blood specimens were collected on the morning after admission following an 8 hour overnight fast. Postprandial C-peptide and insulin samples were obtained at 1 hour and 2 hours after a standardized inpatient breakfast containing approximately 50–60 g of carbohydrates, consistent with the standard protocol for routine clinical postprandial glucose testing. All blood samples were collected and tested upon admission. The Homeostasis Model Assessment 2 (HOMA2) was used to calculate the β-cell function index and insulin resistance index. HOMA2-β,HOMA2-IR and HOMA2-S were calculated based on fasting insulin, while indices derived from fasting C-peptide were used for sensitivity analyses. Calculations were performed using the HOMA2 Calculator provided by the University of Oxford Diabetes Trials Unit(https://www.dtu.ox.ac.uk/homacalculator/).

### 2.3 Continuous glucose monitoring parameters

A subcutaneous interstitial fluid glucose monitoring system (Medtronic) was utilized. All participants were fitted with the sensor on the day of admission. The system generates 288 glucose readings per 24 hours(one measurement every 5 minutes). Data from the first 3 consecutive days after admission were used for the final analysis.

Stringent data quality control rules were predefined and implemented during data cleaning. A minimum valid data coverage rate of 70% within the 3-day analytical window was set as the inclusion threshold. Participants were excluded if their data coverage fell below this threshold due to sensor displacement, device hardware failure, or signal interruption. All subjects included in the final analysis met the 70% data completeness requirement, ensuring the reliability of all CGM-derived metrics. The primary outcome, time in range (TIR), was calculated based on all valid CGM readings within the 72-hour monitoring window. The target glucose range was defined as 3.9–10.0 mmol/L, in accordance with the international consensus on clinical CGM application. TIR was computed as the percentage of total valid monitoring time spent within the target glucose range, ensuring comparability of the outcome across all participants. The average data from the three days prior to admission were used for analysis. The collected CGM metrics included: TIR (3.9–10.0 mmol/L), time above range (TAR; Level 1: >10.0 mmol/L, Level 2: >13.9 mmol/L), time below range (TBR; Level 1: <3.9 mmol/L, Level 2: <3.0 mmol/L), mean blood glucose (MBG), standard deviation of blood glucose (SDBG), mean amplitude of glycemic excursions (MAGE), glucose management indicator (GMI), largest amplitude of glycemic excursions (LAGE), and coefficient of variation (CV).

### 2.4 Statistical analysis

All data analyses were performed using R software (version 4.5.1; R Foundation for Statistical Computing, Vienna, Austria) with the ‘rms’ package for restricted cubic spline analysis and ‘lm’ package for linear regression.). All tests were two-sided, and a P-value < 0.05 was considered statistically significant. Continuous variables were evaluated for normal distribution using the Shapiro-Wilk test combined with Q-Q plots. Normally distributed continuous variables are presented as mean ± standard deviation (SD), and between-group comparisons were performed using one-way analysis of variance (ANOVA). Non-normally distributed variables are presented as median (25th–75th percentiles), with between-group comparisons conducted using the Kruskal-Wallis H test. Categorical variables are expressed as numbers (percentages), and the Chi-square (χ^2^) test was utilized for comparisons.

Subjects were stratified into three groups based on TIR levels: Group 1 (TIR <70%), Group 2 (70% ≤ TIR <85%), and Group 3 (TIR ≥85%).

To evaluate the independent association between TIR and islet function, multiple linear regression analysis was conducted with TIR as a continuous dependent variable. Variables demonstrating significant differences in between-group comparisons were first subjected to univariate correlation analysis to identify potential covariates. Variables with P < 0.05 in univariate analysis were subsequently incorporated into the multiple regression models. HOMA2-β was assigned as the core independent variable, and potential confounding factors were adjusted in a stepwise manner. Results are presented as regression coefficients (β) and their 95% confidence intervals (CIs).

Additionally, ordinal logistic regression was performed as a sensitivity analysis.

It should be noted that multicollinearity among independent variables was assessed using the variance inflation factor (VIF), with VIF > 10 considered indicative of severe collinearity. Furthermore, because the calculation formula for HOMA2-β directly includes fasting plasma glucose (FPG), there is a mathematical coupling between them. To evaluate whether adjusting for FPG would lead to overadjustment, we constructed two nested multiple linear regression models (Model A: unadjusted for FPG and HbA1c; Model B: adjusted for FPG and HbA1c). Ultimately, it was decided not to include FPG or HbA1c in the linear multiple correlation analysis.

To further explore whether a nonlinear relationship exists between pancreatic islet function and TIR, a restricted cubic spline (RCS) analysis was performed. Based on the multiple linear regression models described above, RCS analysis was conducted for the association between the core variable HOMA2-β and TIR, adjusting for the same covariates, allowing the association to be presented as a smooth curve. The significance of the nonlinear relationship was assessed using the likelihood ratio test.

Finally, sensitivity analyses were performed to test the robustness of the main conclusions. To address potential confounding by concomitant hypoglycemic agents, chi-square tests were first conducted to assess the balance of treatment regimens across TIR strata. To further evaluate the potential impact of hypoglycemic agent heterogeneity on the core conclusions, a medication-restricted sensitivity analysis was performed in two predefined homogeneous subgroups based on baseline treatment regimens: (1) drug-naive patients with no prior glucose-lowering treatment; (2) patients receiving only metformin monotherapy. Patients with exposure to GLP-1 receptor agonists, DPP-4 inhibitors, SGLT2 inhibitors, sulfonylureas, insulin, or thiazolidinediones were excluded from both subgroups. This sensitivity analysis followed exactly the same statistical strategy as the primary analysis. Restricted cubic spline (RCS) regression was applied to model the nonlinear relationship between HOMA2-*β* and TIR, with identical knot placement, covariate adjustment set (age, BMI, HOMA2-IR, and duration of diabetes), and nonlinearity testing method as the primary analysis. Only the analytical population was replaced to rule out methodological differences as a confounder of the results. A medication-restricted sensitivity analysis was further performed, including only drug-naive patients and patients receiving metformin monotherapy, to eliminate potential interference from other glucose-lowering drugs on the core association between HOMA2-*β* and TIR.

Correlation analyses between HOMA2-β and HOMA2-IR were conducted across TIR subgroups to explore possible explanations for the nonlinear relationship between islet function and TIR. To investigate whether TIR (as a continuous or categorical variable) moderates the relationship between HOMA2-β and HOMA2-IR, interaction analyses were performed. Given that the primary analysis used HOMA2-β calculated from fasting insulin, all analyses were repeated with the core indicator replaced by HOMA2-β calculated from fasting C-peptide, and the consistency of the association pattern and statistical significance before and after the replacement was compared.

## 3 Results

### 3.1 Demographic characteristics

All of the 1,204 initial T2DM patients meeting the inclusion and exclusion criteria, 44 were excluded due to incomplete clinical data, leaving a final cohort of 1,160 patients. The baseline characteristics are summarized in **Table 1**. The cohort included 695 men (59.9%), with a mean age of 52.3 ± 12.8 years. The median duration of diabetes was 3.0 (0.5–7.0) years, and the mean BMI was 26.25 ± 4.00 kg/m^2^. At admission, the median FPG was 8.56 (7.10–11.10) mmol/L, median FCP was 1.87 (1.36–2.53) ng/mL, median FINS was 9.82 (6.47–14.43) μU/mL, and median HbA1c was 8.0% (6.90%–9.60%).

### 3.2 Between-group comparisons

**Figure 1:**
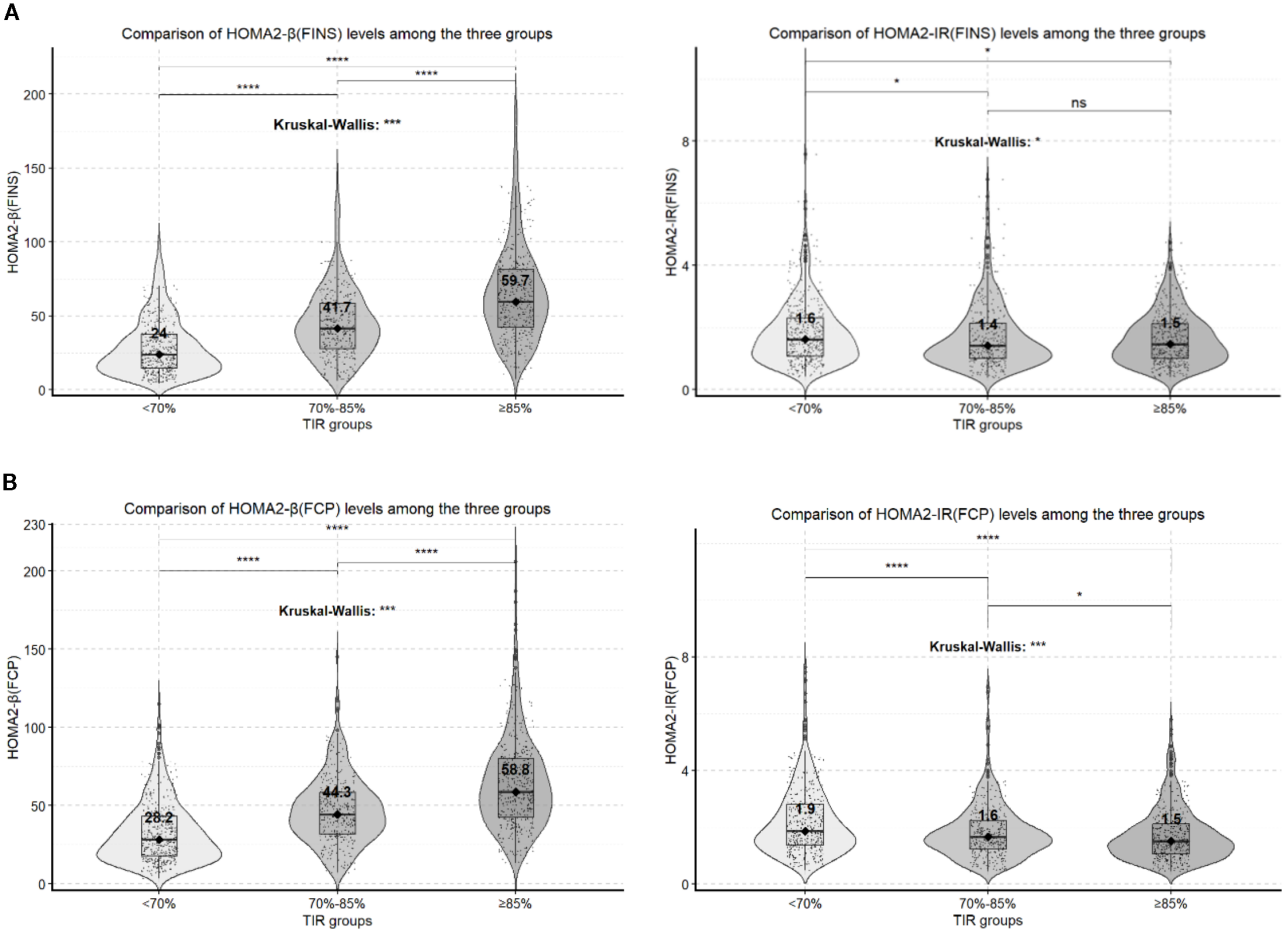
**(A)** Shows HOMA2-IR(FINS) and HOMA2-*β* (FINS) across TIR groups. HOMA2-IR(FINS) was highest in the TIR <70% group and decreased with increasing TIR, with significant differences between the <70% and ≥85% groups (P < 0.05). HOMA2-*β* (FINS) increased progressively with higher TIR, with significant differences between all pairwise comparisons (****P < 0.001 for <70% vs 70–85%; **P < 0.01 for <70% vs ≥85%; *P < 0.05 for 70–85% vs ≥85%). Alt text: shows HOMA2-IR(FINS) and HOMA2-*β* (FINS) across TIR groups. HOMA2-IR(FINS) was highest in the TIR <70% group and decreased with increasing TIR, with significant differences between the <70% and ≥85% groups (P < 0.05). HOMA2-*β* (FINS) increased progressively with higher TIR, with significant differences between all pairwise comparisons (***P < 0.001 for <70% vs 70–85%; **P < 0.01 for <70% vs ≥85%; *P < 0.05 for 70–85% vs ≥85%).Alt text: Violin plot displaying the distribution of HOMA2-IR(FINS) and HOMA2-*β* (FINS) across three TIR subgroups (TIR<70%, 70%≤TIR<85%, TIR≥85%); HOMA2-IR(FINS) was the highest in the TIR<70% group and decreased with elevated TIR with a significant difference between TIR<70% and TIR≥85% groups (P<0.05), while HOMA2-*β* (FINS) increased progressively with higher TIR and all pairwise comparisons showed statistically significant differences (P<0.001 for <70% vs 70–85%, P<0.001 for <70% vs ≥85%, P<0.01 for 70–85% vs ≥85%). **(B)** Shows HOMA2-IR(FCP) and HOMA2-*β* (FCP) across TIR groups. HOMA2-IR(FCP) showed a significant decreasing trend with increasing TIR, with significant differences between all pairwise comparisons (<70% vs 70–85%: **P < 0.001; <70% vs ≥85%: *P < 0.01; 70–85% vs ≥85%: ****P < 0.0001). HOMA2-*β* (FCP) increased progressively with higher TIR, with significant differences between all pairwise comparisons (<70% vs 70–85%: **P < 0.001; <70% vs ≥85%: *P < 0.01; 70–85% vs ≥85%: ****P < 0.0001).

Participants were divided into three groups according to TIR: TIR <70%, 70% ≤ TIR <85%, and TIR ≥85%. Between-group comparisons revealed significant differences across the TIR groups in age, sex, BMI, FPG, HbA1c, 1-hour and 2-hour postprandial C-peptide, 1-hour and 2-hour postprandial insulin, HOMA2-*β* (FINS), HOMA2-IR (FINS), HOMA2-*β* (FCP), HOMA2-IR (FCP), TG, TC, HDL-C, eGFR, serum uric acid, presence of fatty liver, TAR, TBR, CV, LAGE, MAGE, GMI, SDBG, and MBG (all P < 0.05). Conversely, no significant differences were observed regarding diabetes duration, history of coronary heart disease, family history, alcohol consumption, hypertension history, treatment history, fasting C-peptide, fasting insulin, AST, ALT, LDL-C, serum creatinine, BUN, and the presence of lower extremity or carotid arterial plaques (all P > 0.05). Preliminary analysis indicated that HOMA2-*β* showed an upward trend as TIR levels increased (**Table 2**; **Figure 1**). Variables with significant between-group differences were considered potential confounders and included in subsequent multivariate analyses.

**Table 2:** Between-group differences among TIR-stratified participants.

Characteristics	TIR<70%	70%≤TIR<85%	TIR>85%	x2/H	Pvalue
N	414	334	412		
Male sex, n (%)	267 (64.5)	187 (55.9)	241 (58.4)	6.589	0.037
Age (year)	51.11 ± 13.19	54.27 ± 12.74	51.94 ± 12.17	6.21	0.002
BMI	26.64 ± 4.12	26.06 ± 3.72	26.02 ± 4.00	3.225	0.04
CD (year)	3.0 (0.29-8.0)	3.0 (0.5-7.0)	3.0 (0.5-7.0)	0.332	0.847
SH, n (%)				8.459	0.206
No	291 (70.4)	255 (76.4)	315 (76.4)		
<10years	40 (9.6)	22 (6.6)	36 (8.8)		
≥10years	83 (20.0)	57 (17)	61 (14.8)		
DH, n (%)	104 (25.20)	77 (23.1)	100 (24.3)	3.049	0.55
CHD, n (%)	59 (14.20)	58 (17.3)	69 (16.8)	3.01	0.56
FH of DM, n (%)	187 (45.2)	140 (41.8)	177 (43.0)	0.917	0.632
HTN,n (%)				7.695	0.464
No	242 (58.5)	184 (55.0)	237 (57.5)		
Grade 1	37 (8.9)	28 (8.4)	30 (7.4)		
Grade 2	58 (14.0)	41 (12.4)	67 (15.7)		
Grade 3	77 (18.6)	81 (24.2)	80 (19.4)		
TH,n (%)				15.93	0.43
Untreated	134 (32.4)	93 (27.9)	130 (31.5)		
Met	131 (31.7)	107 (32)	107 (25.9)		
Met+others	53 (12.8)	60 (17.9)	59 (14.3)		
Others	96 (23.1)	68 (20.5)	116 (28.3)		
HbA1c	9.40 (8.18-11.2)	7.80 (7.00-9.09)	6.9 (6.2-8.0)	357.98	<0.001
FPG	11.49 (8.91-14.7)	8.49 (7.19-10.24)	7.27 (6.31-8.52)	392.62	<0.001
FCP	1.9 (1.34-2.60)	1.87 (1.39-2.50)	1.85 (1.38-2.52)	0.264	0.876
FINS	9.77 (6.41-14.1)	9.15 (6.19-14.37)	10.39 (6.68-14.8)	4.633	0.099
P1hCP	2.75 (1.97-4.09)	3.27 (2.35-4.44)	3.70 (2.39-5.17)	38.38	<0.001
P1hINS	27.56 (17.1-41.06)	30.36 (21.1-47.67)	34.59 (23.0-54.5)	32.99	<0.001
P2hCP	3.46 (2.46-5.26)	4.47 (3.10-6.24)	5.00 (3.31-7.10)	71.53	<0.001
P2hINS	31.94 (19.97-46.79)	35.4 (24.66-54.34)	38.8 (26.58-63.3)	31.14	<0.001
TG	2.53 (1.67-2.48)	2.08 (1.41-2.97)	1.91 (1.33-2.88)	45.99	<0.001
TC	5.17 (4.39-6.06)	4.93 (4.16-5.64)	4.96 (4.25-5.76)	13.13	0.01
HDL-C	1.114 ± 0.358	1.22 ± 0.66	1.22 ± 0.33	12.44	<0.001
LDL-C	3.021 ± 0.934	2.88 ± 0.88	2.92 ± 1.01	2.36	0.83
SCr	64.65 ± 24.52	64.01 ± 17.08	67.02 ± 19.16	2.27	0.104
SUA	331.67 ± 101.42	326.56 ± 86.75	343.57 ± 92.00	3.53	0.03
BUN	6.02 ± 1.86	6.04 ± 1.68	6.01 ± 1.63	0.03	0.969
eGFR	104.70 ± 18.69	102.08 ± 15.83	101.07 ± 16.33	4.48	0.012
ALT (U/L)	27 (18-44)	24.50 (19.0-36.0)	24 (17-40)	7.57	0.23
AST (U/L)	21 (17-32))	21 (17.0-27.0)	21 (17-29)	0.54	0.764
TAR CV	47 (37-68) 22.59 ± 5.94	21 (18-25) 24.59 ± 5.77	7 (3-11) 21.59 ± 4.72	1041.5 29.2	<0.001 <0.001
LAGE	13.07 ± 2.85	11.22 ± 2.61	8.90 ± 2.34	275.08	<0.001
GMI	7.7 (40-8.30)	6.9 (6.8-7.1)	6.4 (6.2-6.6)	957.71	<0.001
MAGE	6.00 (4.80-7.35)	5.1 (4.1-6.2)	3.8 (3.2-4.6)	348.84	<0.001
SDBG	2.70 (2.30-3.10)	2.2 (1.90-2.50)	1.7 (1.4-1.9)	599.65	<0.001
MBG	10.66 ± 1.61	8.35 ± 0.61	7.11 ± 0.65	1178.7	<0.001
FLD	377 (91.1)	275 (82.42)	336 (81.5)	15.06	<0.001
CAP	327 (78.9)	261 (78.1)	317 (76.9)	0.39	0.822
LEAP	228 (55.0)	178 (53.4)	214 (52.0)	0.66	0.719
HOMA2-β (FINS)	24 (14.50-38.33)	41.7 (27.60-59.2)	59.7 (42.35-81.6)	320.32	<0.001
HOMA2-IR (FINS)	1.6 (1.08-2.30)	1.4 (0.99-2.14)	1.5 (1.00-2.12)	6.63	0.036
HOMA2-β (FCP)	28.2 (17.95-43.40)	44.3 (31.85-58.5)	58.8 (42.38-80.05)	302.07	<0.001
HOMA2-IR (FCP)	1.89 (1.36-2.84)	1.6 (1.22-2.25)	1.5 (1.05-2.11)	43.45	<0.001

Data are mean ± SD, median (IQR), or n (%). Intergroup comparisons used Kruskal-Wallis H test or χ^2^ test; P<0.05 was considered statistically significant.

T2DM, type 2 diabetes mellitus; TIR, time in range; BMI, body mass index; CD, History of drink; SH, History of smoke; CHD, History of coronary heart disease; FH of DM, Family history of diabetes; HDN, History of hypertension; TH, Treatment history; Met, Metformin monotherapy; Met+others, Metformin and other oral hypoglycemic agents; others, other medications; FPG FCP, Fasting C-peptide; FINS, Fasting insulin; P1hCP, 1-hour postprandial C-peptide; P1hINS, 1-hour postprandial insulin; P2hCP, 2-hour postprandial C-peptide; P2hINS, 2-hour postprandial insulin; TG, Triglyceride; TC, Cholesterol; HDL-C, High-density lipoprotein cholesterol; LDL-C, Low-density lipoprotein; SCr, Serum creatinine; SUA, Serum uric acid; BUN Urea; ALT, Alanine Aminotransferase; AST, Aspartate Aminotransferase; TAR, Time Above Range; CV, Coefficient of Variation; LAGE, Largest Amplitude of Glycemic Excursions; GMI, glucose management indicator; MAGE, Mean Amplitude of Glycemic Excursions; SDBG, Standard Deviation of Blood Glucose; MBG, Mean Blood Glucose; FLD, Non-alcoholic fatty liver; CAP, Carotid artery plaque; LEAP, Lower extremity arterial plaque; HOMA2-*β* (FINS), HOMA2-*β* (based on FINS); HOMA2-IR (FINS), HOMA2-IR (based on FINS); HOMA2-*β* (FCP), HOMA2-*β* (based on FCP); HOMA2-IR (FCP), HOMA2-IR (based on FCP).

### 3.3 Univariate correlation analysis

Spearman rank correlation analysis was performed to identify confounders for the multivariate analysis. The core variable, HOMA2-*β* (FINS), demonstrated a significant positive correlation with TIR (r = 0.480, 95% CI: 0.435–0.523, P < 0.001), while HOMA2-IR (FINS) showed a significant negative correlation with TIR *r* = −0.131, 95% *CI*: −0.187 *to* −0.074, *P* < 0.001. Similarly, HOMA2-*β* (FCP) positively correlated with TIR *r* = 0.495, *P* < 0.001, and HOMA2-IR (FCP) negatively correlated with TIR *r* = −0.087, *P* = 0.003. Both FPG and HbA1c were strongly and negatively correlated with TIR (r = -0.665 and r = -0.572, respectively, both P < 0.001). Furthermore, 1-hour and 2-hour postprandial C-peptide, 1-hour and 2-hour postprandial insulin, and HDL-C were positively correlated with TIR in univariate analysis (all P < 0.05). These crude correlations reflect the overall trend across the full cohort, where higher postprandial insulin levels are generally accompanied by better overall islet reserve and better glycemic control. Diabetes duration, LDL-C, SCr, SUA, and BUN showed no significant correlation with TIR (all P > 0.05). These analyses suggest that stronger basal islet *β*-cell function is associated with longer time spent in the target glucose range.

### 3.4 Core association analysis

**Figure 2:**
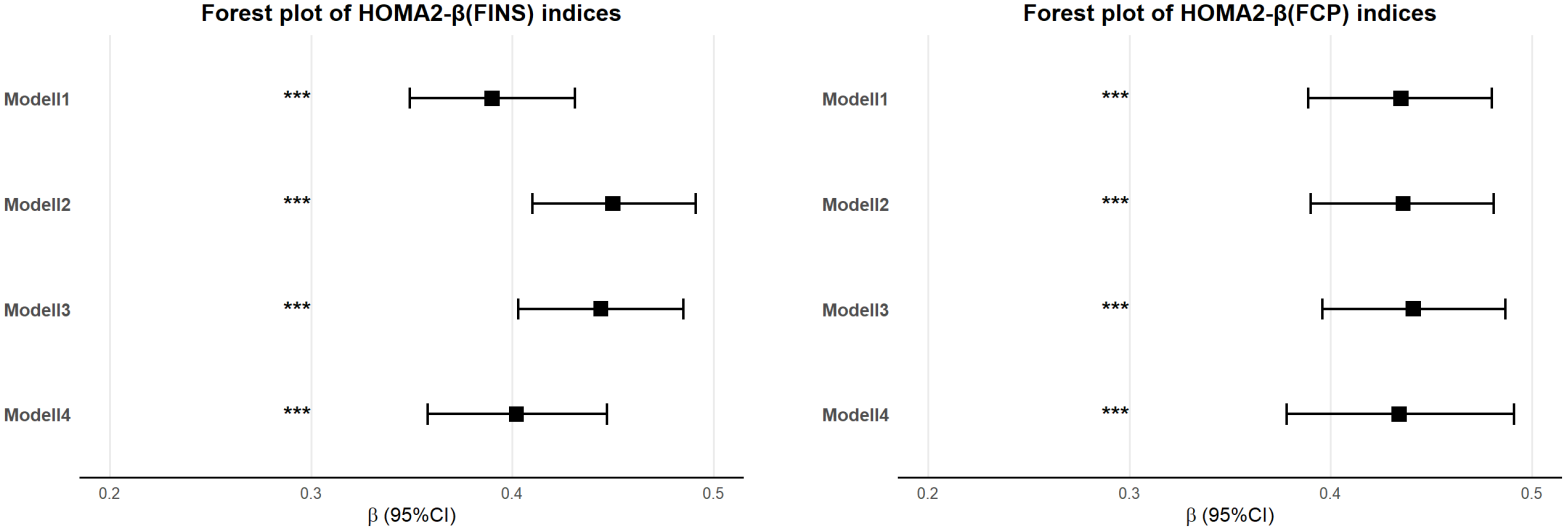
This forest plot was constructed based on the results of **Table 3**, using the regression coefficient (*β*) and its 95% confidence interval (CI) for the core variable. Panel (1) presents the findings derived from fasting insulin (FINS), and Panel (2) those from fasting C-peptide (FCP). It illustrates the association between time in range (TIR) and HOMA2-*β* across four sequentially adjusted models. The blue square at each level denotes the estimated *β* coefficient, with the size of the square reflecting the weight of the estimate; the horizontal line segments represent the 95% CIs.

Based on the univariate analysis, multiple linear regression models were constructed with TIR as a continuous outcome variable (**Table 3**). Model 1 (unadjusted, including only HOMA2-*β* [FINS]) revealed a significant positive association between HOMA2-*β* and TIR (*β* = 0.390, 95% CI: 0.349–0.431, P < 0.001), explaining 25.6% of the variance in TIR *R*^2^ = 0.256. Model 2 incorporated HOMA2-IR (FINS) into Model 1; HOMA2-*β* remained significantly positively associated with TIR *β* = 0.450, *P* < 0.001, while HOMA2-IR showed a significant negative association. Model 3 further adjusted for demographic characteristics, where HOMA2-*β* maintained its positive association *β* = 0.444, *P* < 0.001, and diabetes duration showed a significant negative association *β* = −0.262, *P* = 0.026. Model 4 included postprandial metabolic indicators and other variables. The results confirmed that HOMA2-*β* (FINS) and 2-hour postprandial C-peptide were significantly positively associated with TIR (both P < 0.001), whereas HOMA2-IR (FINS), diabetes duration, 2-hour postprandial insulin, and TG were negatively associated (all P < 0.05). Notably, the direction of association for 2-hour postprandial insulin reversed from positive in univariate analysis to negative in the fully adjusted model. This is because after adjusting for basal *β*-cell function and insulin resistance, higher postprandial insulin at equivalent basal function levels indicates delayed insulin secretion rhythm and reduced insulin secretory efficiency, which corresponds to poorer postprandial glycemic control. In contrast, 2-hour postprandial C-peptide remained positively associated with TIR, consistent with its property of being unaffected by peripheral insulin clearance and more accurately reflecting endogenous insulin secretion capacity. The final model explained 37.4% of the variance *R*^2^ = 0.374. Sensitivity analyses substituting FINS-based HOMA2 with FCP-based HOMA2 yielded consistent conclusions (**Table 3**, Panel 2). A forest plot (**Figure 2**) visually confirms that across all models, the Beta coefficients for HOMA2-*β* remained robustly positive without crossing the null value.

**Table 3:** The relationship between islet function and time in range (TIR).

Variable	β	95% CI	Pvalue	R2	SE
model1					
HOMA2-β (FINS)	0.39	0.349-0.431)	<0.001	0.256	0.021
model2					
HOMA2-β (FINS)	0.45	0.410-0.4911	<0.001	0.329	0.021
HOMA2-IR (FINS)	-6.589	(-7.819--5.539)	<0.001		0.627
model3					
HOMA2-β (FINS)	0.444	(0.403-0.485)	<0.001	0.3335	0.021
HOMA2-IR (FINS)	-6.477	(-7.854--5.109)	<0.001		0.697
Duration of diabetes (year)	-0.262	(-0.496--0.027)	0.026		0.12
Sex/BMI/Age			p>0.05		
model4					
HOMA2-β (FINS)	0.402	(0.358-0.447)	<0.001	0.374	0.023
HOMA2-IR (FINS)	-5.987	(-7.391--4.854)	<0.001		0.175
Duration of diabetes (year)	-0.262	(-0.493--0.028)	0.028		0.118
P2hCP	1.837	(0.748-2.952)	<0.001		0.555
P2hINS	-0.099	(-0.169--0.029)	0.006		0.036
TG	-1.023	(-1.415--0.632)	<0.001		0.2
P1hCP/P1hINS/HDL-C/Sex/Age/BMI/eGFR			p>0.05		
(2) Sensitivity analysis with HOMA2-β (FCP).					
Variable	β	95% CI	Pvalue	R2	SE
model1				0.258	
HOMA2-β (FCP)	0.435	(0.389-0.480)	<0.001		0.023
model2				0.265	
HOMA2-β (FCP)	0.436	(0.390-0.481)	<0.001		0.023
HOMA2-IR (FCP)	-0.566	(-0.917--0.214)	<0.001		0.179
model3				0.287	
HOMA2-β (FCP)	0.441	(0.396-0.487)	<0.001		0.023
HOMA2-IR (FCP)	-0.465	(-0.815--0.116)	<0.001		0.178
BMI	-0.759	(-1.106--0.412)	<0.001		0.177
Sex/Age/Duration of diabetes			>0.05		
model4				0.332	
HOMA2-β (FCP)	0.434	(0.378-0.491)	<0.001		0.029
HOMA2-IR (FCP)	-0.384	(-0.727--0.04)	<0.001		0.175
Duration of diabetes	-0.172	(-0.415--0.007)	0.163		0.124
P2hCP	1.154	(0.371-2.657)	0.009		0.582
P2hINS	-0.073	(-0.145--0.001)	0.048		0.037
TG	-1.112	(-1.519--0.704)	<0.001		0.208
P1hCP/P1hINS/HDL-C/Sex/ Age/BMI/eGFR/Duration of diabetes			>0.05		

(1): Model 1: only HOMA2-*β*; Model 2: adjusted for HOMA2-IR; Model 3: further adjusted for demographic characteristics; Model 4: additionally adjusted for postprandial and metabolic parameters. Data are *β* (95% CI), P value, R^2^,SE. (2): These results represent a sensitivity analysis of (1), verified using core indices calculated based on fasting C-peptide (FCP). In Model 1, only the core independent variable HOMA2-*β* (FCP) was included, and a multiple linear regression analysis was performed with time in range (TIR), the glycemic control index, as the continuous dependent variable. HOMA2-IR (FCP) was adjusted for in Model 2, partial demographic characteristics in Model 3, and laboratory-related indicators in Model 4. All the above models were sequentially adjusted on the basis of the core variable in Model 1.

Collinearity diagnosis and sensitivity analysis: Collinearity diagnosis showed that the variance inflation factors (VIF) for HOMA2-*β*, FPG, HbA1c, and HOMA2-IR were 3.34, 4.53, 2.41, and 1.84, respectively, all <5, indicating no severe multicollinearity. The correlation matrix revealed that TIR was moderately correlated with FPG (r = –0.669), HbA1c (r = –0.577), and HOMA2-*β r* = 0.511. However, in the model without adjustment for FPG and HbA1c, the regression coefficient for HOMA2-*β* with TIR was 0.402 (SE = 0.0194, 95% CI: 0.358–0.447, P < 0.001); after additionally including FPG and HbA1c, the regression coefficient for HOMA2-*β* decreased to 0.079 (SE = 0.0310, 95% CI: 0.018–0.140, P = 0.011), representing an 80.35% reduction, and the standard error increased by 60.2%. These results indicate that, although statistical collinearity was not significant, there is a strong mathematical coupling between HOMA2-*β* and FPG, and HbA1c is highly correlated with glycemic control indicators; adjusting for these variables simultaneously leads to overadjustment. Therefore, FPG and HbA1c were not included in the primary analysis model.

For sensitivity analysis, TIR was treated as a categorical variable and multiple logistic regression analysis was performed. Confounding factors were adjusted sequentially in the same manner to construct models. The results showed that the association between HOMA2-*β* and TIR remained significant. Furthermore, in Model 4 of the logistic regression sensitivity analysis, which adjusted for FPG and HbA1c, although the effect size was attenuated due to collinearity, HOMA2-*β* remained an independent positive predictor of TIR (OR = 1.027, 95% CI: 1.016–1.039, P < 0.001). That is, each 1-unit increase in HOMA2-*β* was associated with a 2.7% increase in the odds of being in a higher TIR category. The test of parallel lines was not significant *P* = 0.991, satisfying the proportional odds assumption. The results are shown in **Table 4**.

**Table 4:** Results of logistic regression analysis.

Model	Variable	β	OR (95% CI)	Pvalue
model1	HOMA2-β (FINS)	0.039	1.040 (1.039-1.049)	<0.001
model2	HOMA2-β (FINS)	0.05	1.055 (1.049-1.062)	<0.001
	HOMA2-IR (FINS)	-0.731	0.48 (0.416-0.556)	<0.001
model3	HOMA2-β (FINS)	0.053	1.052 (1.047, 1.062)	<0.001
	HOMA2-IR (FINS)	-0.72	0.49 (0.404, 0.586)	<0.001
	P2hINS	-0.014	0.99 (0.978, 0.994)	0.001
	P2hCP	0.17	1.19 (1.044, 1.347)	0.009
	Duration of diabetes	-0.035	0.97 (0.942, 0.991)	0.008
	TG	-0.085	0.92 (0.874, 0.965)	<0.001
	BMI/Age/TC/HDL-C/P1hCP/P1hINS/Sex/eGFR			>0.05
model4	HOMA2-β (FINS)	0.027	1.03 (1.016, 1.039)	<0.001
	HOMA-2IR (FINS)	-0.346	0.71 (0.558, 0.897)	0.004
	FPG	-0.147	0.86 (0.780, 0.956)	0.005
	HbAlc	-0.278	0.76 (0.677, 0.848)	<0.001
	TG	-0.101	0.90 (0.852, 0.960)	<0.001
	Duration of diabetes	-0.038	0.96 (0.947, 0.979)	<0.001
	P2hINS	-0.009	0.99 (0.982, 0.999)	0.034
	Age	-0.038	0.96 (0.947, 0.979)	<0.001
	P1hCP/P1hINS/P2hCP/HDL-C/BMI/eGFR			>0.05

In Model 1, only the core independent variable HOMA2-*β* (FINS) was included for an ordinal logistic regression analysis, with TIR stratification (the glycemic control index) as the outcome variable. HOMA2-IR (FINS) was adjusted for in Model 2, partial demographic characteristics in Model 3, and laboratory-related indicators in Model 4. All the above models were sequentially adjusted on the basis of the core variable in Model 1. Parallel line test: *P*=0.991, which supported the proportional odds assumption.

### 3.5 Nonlinear relationship exploration and sensitivity analysis

**Figure 3:**
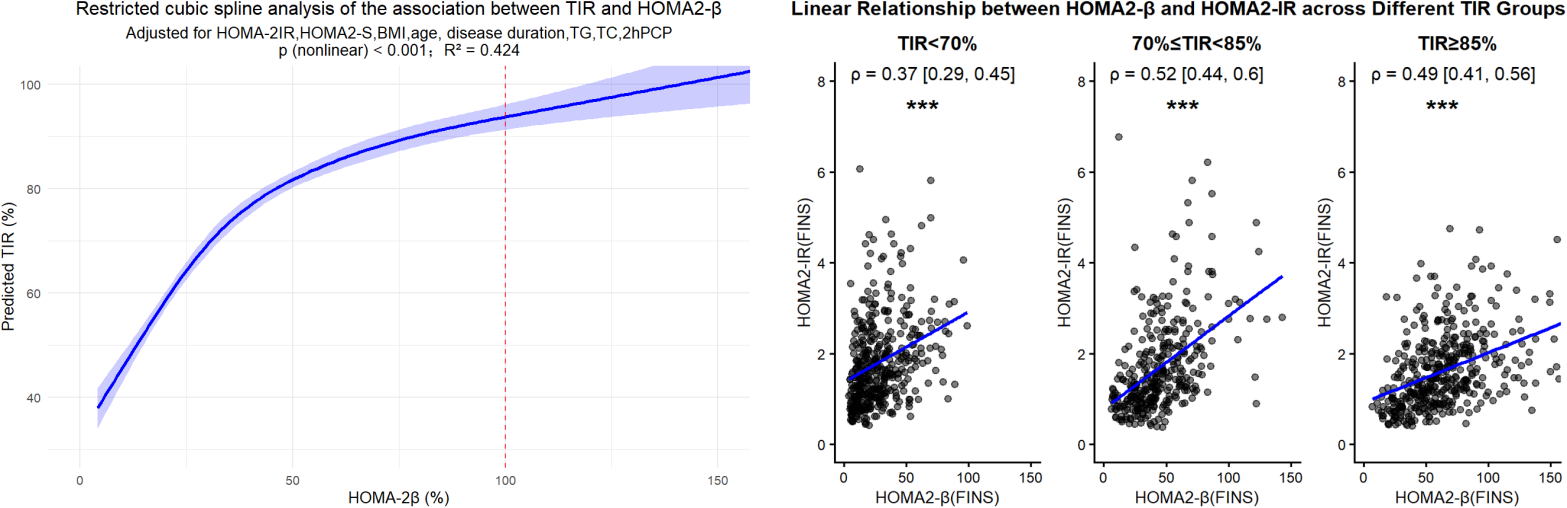
Shows the restricted cubic spline (RCS) curve between HOMA2-*β* (FINS) and TIR after adjusting for HOMA2-IR, HOMA2-S, BMI, age, disease duration, TG, TC, and 2h postprandial C-peptide (2hPCP). The overall trend of the blue fitted curve indicates that TIR increases with rising HOMA2-*β*, but the rate of increase gradually slows. Specifically, when HOMA2-*β* increased from approximately 10% to 100%, the predicted TIR rose rapidly from about 38% to approximately 96%, representing an increase of 58%. The purple shaded area represents the 95% confidence interval. The P value for nonlinearity was <0.001, and the model coefficient of determination (R^2^) was 0.424.

Based on the above analysis, we can conclusively demonstrate that pancreatic islet function exhibits an independent positive correlation with time in range (TIR).To further explore whether a linear relationship exists between HOMA2-*β* and TIR, a restricted cubic spline (RCS) analysis was performed, and the results were visualized using an RCS plot (**Figure 3**). After adjusting for HOMA2-IR (FINS), 2-hour postprandial C-peptide, 2-hour postprandial insulin, disease duration, and triglycerides (TG), a significant nonlinear association was observed between TIR and HOMA2-*β* (FINS) (overall association P < 0.001; P for nonlinearity < 0.001). At lower levels of HOMA2-*β* TIR increased rapidly with rising HOMA2-*β*; however, when HOMA2-*β* exceeded approximately 50%, the increasing trend of TIR markedly slowed. This nonlinear pattern suggests that after HOMA2-*β* reaches a certain threshold, its beneficial effect on glycemic control gradually diminishes. The model explained 42.4% of the variance in TIR *R*^2^ = 0.424.

To further validate the robustness of this nonlinear association and rule out potential confounding by concomitant hypoglycemic agents, a prespecified sensitivity analysis was conducted in the medication-restricted subgroup, which included only drug-naive patients and patients receiving metformin monotherapy. Consistent with the primary full-cohort analysis, the nonlinear relationship between HOMA2-*β* and TIR remained highly statistically significant in this subgroup (P for nonlinearity < 0.001), with an adjusted model R^2^ of 0.383. The shape of the fitted spline curve was highly aligned with that of the primary analysis, showing a typical saturating positive pattern: TIR rose rapidly with improvements in *β*-cell function at lower HOMA2-*β* levels, and the curve gradually plateaued with a markedly reduced increment in TIR once HOMA2-*β* exceeded 100%.These results confirm that the observed nonlinear association between HOMA2-*β* and TIR is independent of the confounding effects of other glucose-lowering medications, supporting the good robustness of the core findings. The fitted RCS curve for the medication-restricted subgroup is provided in **Supplementary Figure 3**, and the baseline characteristics of this subgroup are summarized in **Supplementary Table 2**.

Subgroup correlation analysis (Spearman’s rank correlation) between HOMA2-*β* and HOMA2-IR showed significant positive correlations in all three groups: TIR < 70% group (*ρ* = 0.37, 95% CI: 0.29–0.45, P < 0.001), 70% ≤ TIR < 85% group (*ρ* = 0.52, 95% CI: 0.44–0.60, P < 0.001), and TIR ≥ 85% group (*ρ* = 0.49, 95% CI: 0.41–0.56, P < 0.001). Overall, the correlation was stronger in the higher TIR groups.

Figure A presents the results based on fasting insulin, and Figure B based on fasting C-peptide. Interaction analysis revealed no significant differences in the slopes of the log-linear relationship between HOMA2-IR and HOMA2-*β* across different TIR levels (interaction term P > 0.05), indicating that TIR did not significantly moderate the association between HOMA2-IR and HOMA2-*β*.

## 4 Discussion

In this cross-sectional study, we demonstrated a significant positive association between basal β-cell function, assessed by the homeostasis model assessment HOMA2−β, and time in range (TIR) derived from continuous glucose monitoring. Multiple linear regression analysis showed a robust positive association between islet function and TIR, which remained significant after comprehensive adjustment for potential confounding factors (52). In this study, although the VIF value indicated no severe collinearity between HOMA2-β and FPG in the traditional sense *VIF* = 3.33, adjusting for FPG reduced the effect estimate of HOMA2-β by more than 80% and increased the standard error by more than 60%. This change was not due to random error but rather to the mathematical definition of HOMA2-β (which depends on FPG). Including two variables that are mathematically interdependent in the same model leads to overadjustment, whereby some of the variance explained by HOMA2-β is erroneously attributed to FPG, thereby attenuating the association between the core exposure (TIR) and the islet function indicator. Therefore, FPG and HbA1c were not included as covariates in the multivariable models in this study.

More importantly, restricted cubic spline analysis revealed a clear nonlinear relationship between the two, rather than a simple linear one. The overall trend was that TIR increased with rising HOMA2-*β*, but the rate of increase gradually slowed. When HOMA2-*β* increased from approximately 10% to 100%, the predicted TIR rose rapidly from about 38% to approximately 96%, an increase of 58%. When HOMA2-*β* continued to increase from 100% to 140%, TIR only slowly increased from 96% to 100%, an increase of only approximately 4%. HOMA2-*β* is a *β*-cell function index calculated from fasting glucose and fasting insulin/C-peptide, with a normal value typically around 100%. Values greater than 100% indicate hyperinsulinemia, while values below 100% indicate decreased *β*-cell function. Theoretically, this index has no upper limit, but values exceeding 100% are generally considered to represent compensatory hyperinsulinemia in the setting of increased insulin resistance. Furthermore, when exploring the relationship between HOMA2-*β* and HOMA2-IR across different TIR levels, higher TIR levels were associated with stronger positive correlations between the two measures. In addition, this study confirmed that TIR was significantly negatively correlated with HOMA2-IR; therefore, greater insulin resistance is associated with a lower probability of achieving TIR targets, which may partially explain the observed nonlinear relationship. Sensitivity analyses using C-peptide-derived HOMA2-*β* yielded consistent conclusions, enhancing the robustness of the findings.

Additionally, we further validated the stability of the core findings through a sensitivity analysis restricted to a medication-defined subgroup (drug-naive patients and those on metformin monotherapy only). The nonlinear saturating association between HOMA2-*β* and TIR remained statistically significant with a nearly identical curve shape after excluding the interference of GLP-1 receptor agonists, SGLT2 inhibitors, and other hypoglycemic agents. This indicates that the observed nonlinear relationship is an inherent biological association driven by *β*-cell function itself, rather than a confounding effect of differential drug distribution across the cohort. This finding is also consistent with the pharmacological mechanisms of these agents: the glucose-dependent insulinotropic effects of GLP-1 receptor agonists exert minimal impact on fasting insulin levels and thus do not systematically bias HOMA2-*β* estimation, accounting for the high consistency between the full-cohort and subgroup analyses.

From a pathophysiological perspective, the saturating pattern of the association may be partly explained by the differential contributions of basal and postprandial insulin secretion to overall glycemic control. HOMA2-*β* captures only basal *β*-cell function under fasting conditions, whereas TIR reflects glycemic control across both fasting and postprandial periods. Once basal insulin secretion reaches a certain threshold, further improvements in postprandial glucose control depend more on incretin-mediated meal-stimulated insulin secretion, insulin sensitivity, and gastric emptying rate, leading to diminishing marginal returns of basal *β*-cell function on TIR. This aligns with existing evidence that postprandial glucose profiles are shaped by the heterogeneity of both insulin secretion and sensitivity in T2DM, and that dynamic insulin secretion pathways independent of basal function play a critical role in postprandial glycemic regulation (44).

The findings of this study have important clinical implications, reinforcing the central role of preserving *β*-cell function in achieving high-quality glycemic control. The observed saturating effect suggests that pursuing glycemic targets alone may not be the optimal strategy in clinical practice. At lower levels of HOMA2-*β*, TIR increases rapidly with improvements in *β*-cell function, indicating that residual *β*-cell secretory capacity may contribute critically to achieving glycemic targets. Therefore, for patients with severely impaired islet function, early introduction of exogenous insulin is advisable to protect residual basal islet function. Greater attention to preserving *β*-cell function in clinical management may be more beneficial for achieving long-term glycemic control and delaying the onset of diabetic complications. For patients with relatively preserved islet function with marked insulin resistance, early implementation of non-pharmacological measures (diet, exercise, weight loss) to reduce insulin resistance may substantially lower the risk of diabetic kidney disease (45). These findings provide a basis for individualized treatment strategies in type 2 diabetes mellitus.

Despite the robust and significant findings, several limitations should be acknowledged. First, the cross-sectional design precludes inference of causal direction. Although impaired *β*-cell function may lead to reduced TIR, poor glycemic control may further impair *β*-cell function through glucotoxicity, creating a bidirectional relationship. Future prospective cohort or interventional studies are needed to elucidate causality. Second, although HOMA2-*β* is suitable for large-scale epidemiological studies, it is calculated from fasting insulin/C-peptide and reflects only basal *β*-cell function, without capturing postprandial or dynamically stimulated secretory capacity. For example, the present study found a positive correlation between 2-hour postprandial C-peptide and TIR, but this information is not incorporated into HOMA2-*β*, indicating that HOMA2-*β* is insufficient for assessing “functional *β*-cell reserve.” Future studies may employ gold-standard methods such as the hyperglycemic clamp technique or intravenous glucose tolerance test, as well as validated dynamic indices derived from oral glucose tolerance tests, for more precise assessment of functional *β*-cell reserve (46).

Third, the 3-day CGM window cannot fully represent long-term glycemic patterns. While a 14-day monitoring period is the established best practice for capturing stable chronic glycemic profiles in outpatient research (47), the 72-hour analytical window was deliberately selected for this inpatient retrospective study based on rigorous methodological and clinical considerations. The rationale is threefold: first, the 14-day standard is designed for long-term outpatient glycemic assessment analogous to HbA1c, whereas our study focuses on the association between basal *β*-cell function and early inpatient glycemic profiles, aligning with the widely adopted short-window paradigm in inpatient CGM research. Second, the early admission window minimizes cumulative confounding from inpatient treatment titration and standardized care, better preserving patients’ native baseline metabolic state. Third, the first 72 hours of hospitalization yields higher data integrity, with lower rates of sensor displacement and missing data than later hospital stays. Notably, accumulated evidence validates the analytical reliability of 3-day CGM: it achieves high consistency with 14-day monitoring for core glycemic metrics (48), effectively characterizes glycemic variability and hypoglycemic events (49), and has been confirmed suitable for baseline metabolic assessment in both Chinese populations (50) and inpatient settings (51). Nevertheless, the relatively short monitoring duration remains a notable limitation, and extrapolation of findings to long-term glycemic outcomes should be made with caution. Additionally, the lack of detailed data on diet, physical activity, and medication adherence introduces potential unmeasured confounding. Additionally, the lack of data on diet, physical activity, and medication adherence constitutes unmeasured confounding. Fourth, this study was based on data from a single center, which may limit the generalizability of the findings to other clinical settings and patient populations. Our exclusion criteria excluded patients treated with insulin, sulfonylureas, and thiazolidinediones, meaning the results may not be directly extrapolated to patients receiving these common glucose-lowering regimens. It should also be noted that our cohort included patients treated with GLP-1 receptor agonists and SGLT2 inhibitors. Although these agents exert minimal interference on the assessment of basal *β*-cell function, and our sensitivity analysis in a medication-restricted subgroup (drug-naive and metformin monotherapy only) confirmed the stability of the core findings, the mild effects of these medications on blood glucose levels cannot be completely eliminated. Additionally, the sample size of the medication-restricted subgroup was smaller than that of the full cohort, resulting in slightly reduced statistical power in the extreme value range of HOMA2-*β*. Nevertheless, the overall association trend remained consistent with the full cohort analysis, and the reliability of the core conclusions was not compromised.

In summary, this study provides evidence of a significant nonlinear association between pancreatic *β*-cell function and TIR in patients with type 2 diabetes mellitus. Our findings emphasize that the patient’s underlying islet functional status should be considered when interpreting TIR. However, improving or restoring *β*-cell function remains a recognized clinical challenge. Therefore, for patients with impaired islet function, timely use of CGM data to capture glycemic fluctuations and the development of individualized treatment plans based on monitoring results may help improve clinical outcomes. For patients with relatively preserved islet function, greater attention may be directed toward other aspects of management, such as improving insulin resistance through diet and exercise.

## Data Availability

The data analyzed in this study is subject to the following licenses/restrictions: The raw clinical data analyzed in this study are derived from the electronic medical record system of The Second Hospital of Jilin University. Due to the restrictions of patient privacy protection and hospital data management regulations, the original individual-level dataset cannot be publicly shared. De-identified aggregated data that support the findings are available from the corresponding author upon reasonable request. Requests to access these datasets should be directed to linn24@mails.jlu.edu.cn.
