# Supplementary material for: Association between islet β-cell function and time in range in patients with type 2 diabetes mellitus: a retrospective cross-sectional study
Source: Front Endocrinol (Lausanne). 2026 Aug 5;17:1901834. doi: 10.3389/fendo.2026.1901834 (PMC13485555; doi:10.3389/fendo.2026.1901834)

Supplementary Material

Restricted Cubic Spline Analysis of the Association between TIR and HOMA2-β in Drug-Naïve or Metformin-Monotherapy Patients: A Sensitivity Analysis

1. Background and Objective

In the primary analysis, we observed a significant non-linear association between HOMA2-β (a marker of pancreatic β-cell function) and time-in-range (TIR) in the overall cohort. To exclude potential confounding by heterogeneous glucose-lowering therapies, we conducted a sensitivity analysis restricted to drug-naïve patients and those receiving metformin monotherapy only. This subgroup represents a more homogeneous population in which the direct effect of β-cell function on glycaemic control can be assessed with minimal interference from insulin secretagogues or exogenous insulin.

2. Methods

2.1. Study Population

From the original dataset, we selected participants who were either untreated (no antidiabetic medication) or treated with metformin alone. All other exclusion criteria were identical to the main analysis.

2.2. Variables and Definitions

Outcome: TIR (%) measured by continuous glucose monitoring.

Exposure: HOMA2-β (%), calculated from fasting C-peptide and glucose using the HOMA2 model.

Covariates: age (years), BMI (kg/m^2), HOMA2-IR (index of insulin resistance), and disease duration (years).

2.3. Statistical Analysis

We employed a restricted cubic spline (RCS) regression with 4 knots placed at the 5th, 35th, 65th, and 95th percentiles of the HOMA2-β distribution. The reference point was set at HOMA2-β = 100% (normal β-cell function). The model was adjusted for all covariates listed above using ordinary least squares (ols function in the rms package of R).

Non-linearity was formally tested by the Wald test for the spline terms. The coefficient of determination (R^2) and the partial F-tests were extracted from the model. All analyses were performed in R (version 4.x) with the packages rms, ggplot2, and

readxl.

3. Results

3.1. Model Fit and Covariate Effects

The overall regression was highly significant ($F = 59.96$, df = 7,675, $P < 0.0001$), with an R^2 of 0.383 (derived from model statistics). Among covariates, HOMA2-IR showed a strong inverse association with TIR (effect = –7.97 per 1.205-unit increase, $P < 0.0001$), while disease duration exhibited a marginal negative trend ($P = 0.064$). Age and BMI were not significant in this subgroup ($P > 0.5$).

3.2. Non-linear Association between HOMA2-β and TIR

The test for non-linearity of the spline term was highly significant ($P < 0.0001$), confirming that the relationship departs from linearity.

The adjusted predicted TIR values as a function of HOMA2-β are depicted in Supplementary Figure S1. The curve demonstrates a steep positive slope when HOMA2-β increases from the lower range (~20%) to approximately 60%, with a mean effect of 24.13 units of TIR per 37.85-unit rise in HOMA2-β. Beyond 60%, the curve gradually flattens and reaches a plateau near 100% (predicted TIR ≈ 98–99%). At the reference point of 100%, the predicted TIR is approximately 99%, and further elevations of HOMA2-β up to the maximum observed value produce negligible additional gains in TIR.

3.3. Graphical Summary

The RCS curve (blue line) with 95% confidence bands (shaded area) is shown below. A vertical red dashed line marks the reference (100%). The x-axis is truncated at 0–150% and y-axis at 30–100% for clarity.

(Please insert your generated figure here with caption:)

Supplementary Figure S1. Restricted cubic spline regression of TIR on HOMA2-β, adjusted for age, BMI, HOMA2-IR, and disease duration, in drug-naïve and metformin-only patients. The reference point is HOMA2-β = 100% (red dashed line). Shaded area = 95% CI. Non-linearity $P < 0.001$; $R^2 = 0.383$.

4. Interpretation and Conclusion

This sensitivity analysis, restricted to a therapeutically homogeneous subgroup, confirms the primary finding of a significant non-linear positive association between β-cell function and glycaemic control (TIR). The plateau effect beyond the normal range (≈100%) suggests a functional ceiling—once β-cell function is preserved to

near-normal levels, further increments do not translate into clinically meaningful TIR improvements.

The robust inverse relationship with HOMA2-IR underscores the complementary role of insulin resistance in determining TIR, even in this subgroup. These results support the notion that therapeutic strategies aiming to restore β-cell function to the normal range, rather than supranormal levels, may be sufficient for optimal glucose control, and that managing insulin resistance remains equally critical.

The sensitivity analysis strengthens the validity of our main conclusions by eliminating potential confounding from diverse medication classes and provides a more precise estimate of the pure β-cell function-TIR relationship in early-stage or metformin-treated patients.

Note to editors/reviewers: The figure and raw data are available upon request. All statistical codes are provided in the main manuscript's supplementary file.

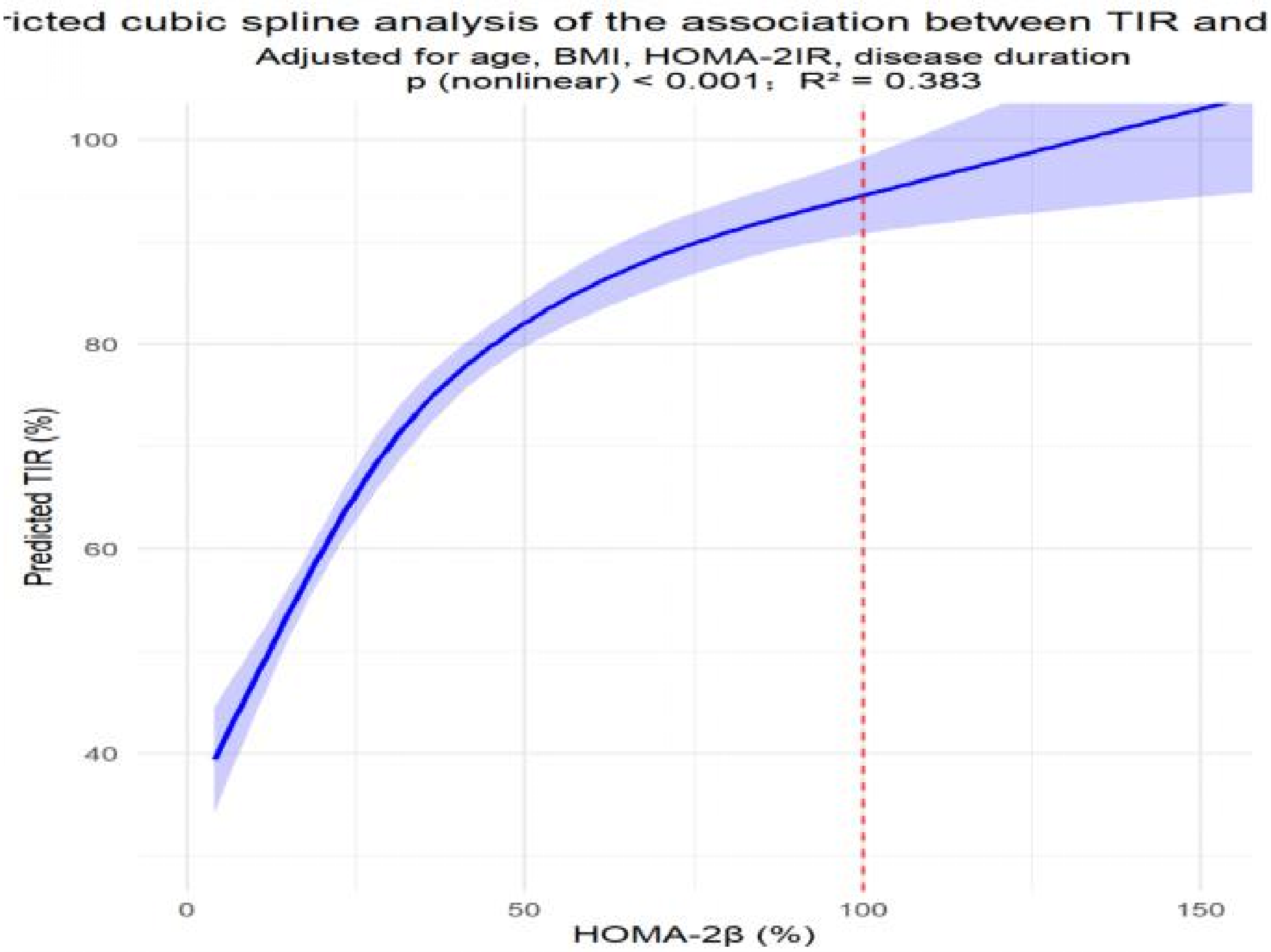

Supplement: Supplementary file 1 [file DataSheet1.pdf]
